# Effects of mechanical stimulation on the reprogramming of somatic cells into human-induced pluripotent stem cells

**DOI:** 10.1186/s13287-017-0594-2

**Published:** 2017-06-08

**Authors:** Young Mi Kim, Yun Gyeong Kang, So Hee Park, Myung-Kwan Han, Jae Ho Kim, Ji Won Shin, Jung-Woog Shin

**Affiliations:** 1 Department of Biomedical Engineering, Inje University, Gimhae, Gyeongsangnam-do Republic of Korea; 2 Department of Microbiology, Chonbuk National University Medical School, Jeonju, Jeollabuk-do Republic of Korea; 3 Department of Physiology, School of Medicine, Pusan National University, Yangsan, Gyeongsangnam-do Republic of Korea; 4 Department of Health Science and Technology/Cardiovascular and Metabolic Disease Center/Institute of Aged Life Redesign/UHARC, Gimhae, Gyeongsangnam-do Republic of Korea

**Keywords:** Human-induced pluripotent stem cells, Cell reprogramming, Reprogramming factors, Human dermal fibroblasts, Mechanical stimulation, Equiaxial stretching

## Abstract

**Background:**

Mechanical stimuli play important roles in the proliferation and differentiation of adult stem cells. However, few studies on their effects on induced pluripotent stem cells (iPSCs) have been published.

**Methods:**

Human dermal fibroblasts were seeded onto flexible membrane-bottom plates, and infected with retrovirus expressing the four reprogramming factors OCT4, SOX2, KLF, and c-MYC (OSKM). The cells were subjected to equiaxial stretching (3% or 8% for 2, 4, or 7 days) and seeded on feeder cells (STO). The reprogramming into iPSCs was evaluated by the expression of pluripotent markers, in vitro differentiation into three germ layers, and teratoma formation.

**Results:**

Equiaxial stretching enhanced reprogramming efficiency without affecting the viral transduction rate. iPSCs induced by transduction of four reprogramming factors and application of equiaxial stretching had characteristics typical of iPSCs in terms of pluripotency and differentiation potentials.

**Conclusions:**

This is the first study to show that mechanical stimuli can increase reprogramming efficiency. However, it did not enhance the infection rate, indicating that mechanical stimuli, defined as stretching in this study, have positive effects on reprogramming rather than on infection. Additional studies should evaluate the mechanism underlying the modulation of reprogramming of somatic cells into iPSCs.

## Background

The first preparation of induced pluripotent stem cells (iPSCs) by Takahashi and Yamanaka in 2006 [1] contributed greatly to the resolution of some of the ethical problems and other difficulties in acquiring embryonic stem (ES) cells [2, 3]. A lot of studies on iPSCs have shown their tremendous potential, demonstrating that the major characteristics and differentiability of iPSCs are almost the same as those of ES cells [4–6]. Recently, much attention has focused on iPSCs for possible uses, such as disease modeling, drug screening, studying pathological states, and other basic studies [7].

However, much remains to be clarified to achieve a routine, safe, and efficacious application of these cells in a clinical context. One current safety problem is the possibility of tumor formation. The oncogene c-MYC has been known to be involved in tumor formation in chimeric mouse-derived iPSCs [8]. One study showed that only three reprogramming factors (OCT4, SOX2, KLF4), excluding c-MYC, enabled mouse or human somatic cells to be reprogrammed into iPSCs, resulting in a decrease in tumor formation compared to reprogramming with the four factors [9]. However, these safety problems require extensive further investigation and resolution.

Another major issue to be resolved is the low efficiency of iPSC preparation. Nakagawa et al. showed 0.02% efficiency for human iPSCs with four reprogramming factors (OCT4, SOX2, KLF4, and c-MYC (OSKM)) [9]. To improve the efficiency of reprogramming, various methods and techniques have been reported. The use of cocktails consisting of OSKM and ONSL (i.e., OCT4, NANOG, SOX2, and Lin28) and the combination of OCT4, SOX2, and NANOG have been shown to be somewhat effective in improving the reprogramming efficiency of human somatic cells [10, 11].

Treatment with the histone deacetylase inhibitor valproic acid (VPA) after introducing three reprogramming factors (OCT4, SOX2, and KLF4) showed higher efficiency (~1.0%) in reprogramming human fibroblasts [12, 13]. However, ~200-times lower efficiency was obtained when they were treated with VPA followed by the introduction of only OCT4 and SOX4.

Recently, some studies using mechanical stimuli in the field of iPSCs research have been published. The micromechanical environment was found to affect the modulation of iPSCs and ES cells when differentiating into certain cell lineages [14, 15], although no specific mechanism has been determined. However, there are no reports on the effects of mechanical stimuli on reprogramming efficiency.

A previous study classified the reprogramming process into three stages, based on molecular changes: initiation, maturation, and stabilization [16]. In this study, for the first time, we applied mechanical stimulation without biochemical factors to investigate its effects on reprogramming efficiency in the initiation stage.

## Methods

### Preparation of cells and media

Human dermal fibroblast cells, STO cells, and PLAT-GP packaging cells were purchased commercially (ATCC, Manassas, VA, USA). The fibroblast growth medium-2 (FGM-2) Bulletkit (Lonza, Tewkesbury, UK) was used for human dermal fibroblast cultures, whereas Dulbecco’s modified Eagle’s medium (DMEM; Gibco, Waltham, MA, USA) containing Glutamax (2 mM; Gibco), penicillin/streptomycin (100 U/mL; Gibco), and fetal bovine serum (FBS, 10%; Life Technologies, Carlsbad, CA, USA) were used for STO and PLAT-GP packaging cells. DMEM/Ham’s F-12 (DMEM/F12; Gibco) medium for human iPSCs and human ES cells contained knockout serum replacement (SR, 20%; Gibco), Glutamax (2 mM; Gibco), nonessential amino acids (NEAA, 0.1 mM; Gibco), 2-mercaptoethanol (0.1 mM; Gibco), 50 U penicillin/streptomycin, and basic fibroblast growth factor (bFGF, 10 μg/mL; Bio-tech/R&D Systems, Minneapolis, MN, USA).

### Retrovirus infection and iPSC generation

#### Preparation of viruses

Using the Lipofectamine-Plus reagent (Life Technologies), pMXs-green fluorescent protein (GFP), pMXs-OCT4, pMXs-SOX2, pMXs-KLF, and pMXs-c-MYC were transfected into prepared PLAT-GP cells (2.5 × 10^4^ cells/cm^2^) with gag-pol and VSV-G [17]. At 4 h after transfection, the medium was replaced with DMEM with 10% FBS. After 48 and 72 h, the supernatant was collected and filtered through a polyvinylidene fluoride (PVDF) filter (pore size 0.45 μm; EMD Millipore, Billerica, MA, USA). Finally, it was concentrated with a Retro-X concentrator (Clontech Laboratories, Inc., Mountain View, CA, USA) for 24 h based on the protocol provided by the manufacturer. Briefly, the Retro-X was added to the acquired supernatant containing virus in a 1:3 volume ratio. Then the mixture was incubated at 4 °C for a day. After a day they were centrifuged at 1500 × g at 4 °C for 45 min. After removing supernatant we added FGM-2. The volume of FGM-2 added was 1/10 of the original solution during centrifugation.

### Virus infection

Human dermal fibroblast cells were seeded on commercial plates or BioFlex (Flexcell Corp., Hillsborough, NC, USA) at a density of 8 × 10^3^ cells/cm^2^. Then, 1 day after seeding, the concentrated supernatant was added twice, once per day for 2 days. Retro-GFP was infected separately to confirm infection. The four reprogramming factors (Retro-OSKM: pMXs-OCT4, pMXs-SOX2, pMXs-KLF, and pMXs-c-MYC) were infected together. Moreover, 10 μg/mL polybrene (Sigma-Aldrich, St. Louis, MO, USA) was added during the infection. Then, 2 days after infection, the cells were cultured in dermal cell growth medium until they were added to feeder cells.

### Applying virus-infected dermal fibroblasts on feeder cells (STO)

STO cells at passage 8, treated with mitomycin C (10 μg/mL; Sigma-Aldrich), were used as feeder cells. Infected fibroblasts (3.5 × 10^3^ cells/cm^2^) were applied to feeder cells using human ES cell medium. The medium was replaced every day for up to 21 days.

### Mechanical stimulation

**Fig. 1:**
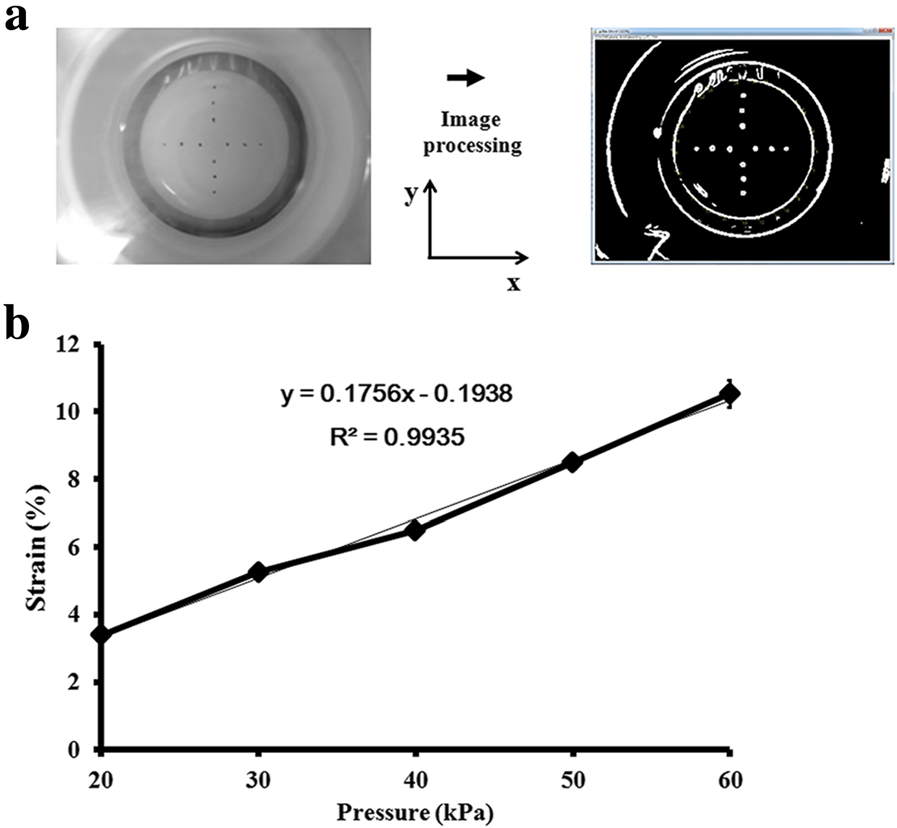
Calibration of pressure versus equiaxial strain. **a** Image processing, (**b**) calibration curve

**Fig. 2:**
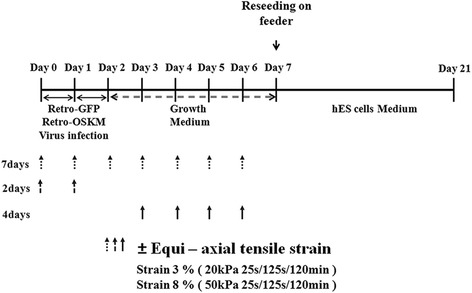
Schematic time schedule for preparing iPSCs. *GRP* green fluorescent protein, *hES* human embryonic stem, *Retro-OSKM* pMXs-OCT4, pMXs-SOX2, pMXs-KLF, pMXs-c-MYC

To provide equiaxial strain, BioFlex plates with a solid cylindrical rod (25 mm in diameter) under each plate (35 mm in diameter) were used. The equiaxial strain magnitude was adjusted by controlling the magnitudes of negative pressures. Through image processing of 12 small particles mounted on the membrane (Fig. 1a), the location of each particle was measured in each pressure step, enabling us to set displacement functions of u(x,y) and v(x,y), where u(x,y) and v(x,y) represent displacement in the x and y directions, respectively. Displacement functions were assumed to be u(x,y) = A + Bx + Cy and v(x,y) = D + Ex + Fy. The coefficients and constants at each pressure step were determined by linear regression based on 12 data points. The origin of the coordinate system was the center of the cylinder rod. The strains in the x and y directions were calculated along shear strains by derivatives. We repeated the measurements at least seven times for each pressure step, which confirmed a uniform equiaxial strain within the cylinder rod. The strains in the x and y directions were similar and the shear strain at each pressure step was negligible (data not shown). Finally, the relationship between pressure and equiaxial strain was obtained by regression analysis and represented as in Fig. 1b. Therefore, the strain magnitude could be controlled by the pressure magnitude. It is worth noting that the cells were carefully seeded from within the rod area. The investigation involved eight groups: CP, FM, FM3%2D, FM3%4D, FM3%7D, FM8%2D, FM8%4D, and FM8%7D. ‘CP’ and ‘FM’ indicate commercial plates and flexible membranes, respectively. The first and second numerical values indicate the strain magnitude (in %) and the duration of stimulation in days (D), respectively. The stimulation pattern was 25 s and 125 s of tension and relaxing, respectively. This was provided for 2 h/day. The entire experimental schedule is shown in Fig. 2, where the schedule for mechanical stimulation in each group is detailed together with the start date and duration of each stimulation.

### Fluorescence-activated cell sorting (FACS)

Retro-GFP virus-infected cells were harvested with trypsin-ethylenediaminetetraacetic acid (EDTA) followed by washing with 0.2% bovine serum albumin (BSA) and 0.09% sodium azide (Sigma-Aldrich) staining buffer. They were then fixed in 1% paraformaldehyde (USB, Fremont, CA, USA). A FACSCanto II flow cytometer (BD Biosciences, Franklin Lakes, NJ, USA) was used, together with the FACSDiva software (ver. 6.1.3).

### Alkaline phosphatase (ALP) staining and immunocytochemistry

ALP staining (Sigma-Aldrich) was performed for 30 min at room temperature in the dark using the Vector Red Alkaline Phosphatase Substrate Kit I (Sigma-Aldrich), in accordance with the manufacturer’s protocol. For immunocytochemical staining, human iPSCs were fixed in 4% paraformaldehyde (USB) in phosphate-buffered saline (PBS) for 10 min at room temperature. After washing twice in PBS, the specimens were blocked with 1% BSA in PBS for 1 h at room temperature. The fixed specimens were incubated with primary antibodies overnight at 4 °C, followed by incubation with secondary antibodies for 1 h at room temperature. The fixed cells were stained with primary antibodies against SSEA-4 (1:100; Abcam, Cambridge, UK), OCT4 (1:100; Cell Signaling, Danvers, MA, USA), SOX2 (1:50; Cell Signaling), NANOG (1:100; Cell Signaling), ALP (1:100; Abcam), and GAPDH (1:10,000; Cell Signaling). The primary antibodies were detected with Alexa Fluor 488 (1:200; Invitrogen, Burlington, Canada) and Alexa Fluor 568 (1:200; Invitrogen) conjugated with secondary antibodies. Images were acquired using a fluorescence microscope (Axio Observer Z1; Carl Zeiss, Oberkochen, Germany).

### Embryoid body formation and differentiation of iPSCs

In vitro differentiation was induced by the formation of embryoid bodies (EBs) as previously described [18]. For EB formation, human iPSCs were harvested by treating with dissociation solution (ReproCELL Incorporated, Japan). The clumps of undifferentiated iPSCs were cultured in DMEM/F12 with 20% SR for 8 days on low-attachment 60-mm plates. The medium was change every other day. After 8 days in suspension culture, floating EBs were seeded again in gelatin-coated dishes in DMEM with 10% FBS for 5 days. The medium was changed every other day. Cells were harvested at day 5 of differentiation. Microscopic images were acquired and analyzed at days 2 to 5 as described above using a light microscope (CK40; Olympus, Tokyo, Japan).

### RNA isolation and analysis of gene expression

Total cellular RNA was extracted using the TRIzol reagent (Ambion Life Technologies, Blijswijk, Netherlands). For reverse-transcription polymerase chain reaction (RT-PCR) analysis, cDNA was synthesized from total RNA (2 μg) using High-Capacity RNA-to-cDNA Master Mix (Applied Biosystems, Foster City, CA, USA). The cDNA was subject to RT-PCR with AccuPower 2XPCR Master Mix (Bioneer Corp, Daejeon, South Korea) and primers targeting the respective genes. PCR products were size-fractionated by 1.2% agarose gel electrophoresis with ethidium bromide (EtBr) and photographed under UV transillumination. RT-PCR analysis was performed for pluripotency and early germ layer markers using the primers listed in Table 1.

**Table 1:** Primers used for reverse-transcription polymerase chain reaction

Target	Primer	Sequence (5′ to 3′)
Total OCT4	F	GAGAAGGATGTGGTCCGAGTGTG
Total OCT4	R	CAGAGGAAAGGACACTGGTCCC
Total SOX2	F	AGAACCCCAAGATGCACAAC
Total SOX2	R	ATGTAGGTCTGCGAGCTGGT
Total KLF4	F	ACCCTGGGTCTTGAGGAAGT
Total KLF4	R	ACGATCGTCTTCCCCTCTTT
Total c-MYC	F	CCTACCCTCTCAACGACAGC
Total c-MYC	R	CTCTGACCTTTTGCCAGGAG
Endo-OCT4	F	GACAGGGGGAGGGGAGGAGCTAGG
Endo-OCT4	R	CTTCCCTCCAACCAGTTGCCCCAAAC
Endo-SOX2	F	GGGAAATGGGAGGGGTGCAAAAGAGG
Endo-SOX2	R	TTGCGTGAGTGTGGATGGGATTGGTG
Endo-KLF4	F	ACGATCGTGGCCCCGGAAAAGGACC
Endo-KLF4	R	TGATTGTAGTGCTTTCTGGCTGGGCTCC
Endo-c-MYC	F	GCGTCCTGGGAAGGGAGATCCGGAGC
Endo-c-MYC4	R	TTGAGGGGCATCGTCGCGGGAGGCTG
hTERT	F	CGGAAGAGTGTCTGGAGCAA
hTERT	R	GGATGAAGCGGAGTCTGGA
TDGF	F	TCCTTCTACGGACGGAACTG
TDGF	R	AGAAATGCCTGAGGAAAGCA
Nanog	F	CAAAGGCAAACAACCCACTT
Nanog	R	ATTGTTCCAGGTCTGGTTGC
pMXs-AS3200	R	TTATCGTCGACCACTGTGCTGCTG
pMXs-L3205	R	CCCTTTTTCTGGAGACTAAATAAA
Exo-hOCT4	F	CCCCAGGGCCCCATTTTGGTACC
Exo-hSOX2	F	GGCACCCCTGGCATGGCTCTTGGCTC
Exo-hKLF4	F	ACGATCGTGGCCCCGGAAAAGGACC
Exo-c-MYC	F	CAACAACCGAAAATGCACCAGCCCCAG
hSOX17	F	CGC TTT CAT GGT GTG GGC TAA GGA CG
hSOX17	R	TAG TTG GGG TGG TCC TGC ATG TGC TG
hAFP	F	GAA TGC TGC AAA CTG ACC ACG CTG GAA C
hAFP	R	TGG CAT TCA AGA GGG TTT TCA GTC TGG A
hCK8	F	CCT GGA AGG GCT GAC CGA CGA GAT CAA
hCK8	R	CTT CCC AGC CAG GCT CTG CAG CTC C
hCK18	F	AGC TCA ACG GGA TCC TGC TGC ACC TTG
hCK18	R	CAC TAT CCG GCG GGT GGT GGT CTT TTG
hMAP2	F	CAG GTG GCG GAC GTG TGA AAA TTG AGA GTG
hMAP2	R	CAC GCT GGA TCT GCC TGG GGA CTG TG
hPAX6	F	ACC CAT TAT CCA GAT GTG TTT GCC CGA G
hPAX6	R	ATG GTG AAG CTG GGC ATA GGC GGC AG

*F* forward, *R* reverse

### Western blots

Total cell protein extracts were obtained using a modified lysis buffer (Cell Signaling, Danvers, MA, USA). Protein concentration was determined according to the Bradford method. Proteins were separated by electrophoresis in a 12% bis-polyacrylamide gel (Bio-Rad, Hercules, CA, USA). Primary antibodies against OCT4, SOX2, KLF4, c-MYC, and GAPDH were used with horseradish peroxidase-conjugated secondary antibodies (all from Cell Signaling). Antibodies bound to the PVDF membrane (EDM Millipore, Billerica, MA, USA) were detected using an ECL chemiluminescent substrate kit (GE Healthcare, Mississauga, ON, USA).

### Teratoma formation and histological analysis

Cells were harvested with dissociation reagent, collected into tubes, and centrifuged; pellets were then suspended in PBS/Matrigel with reduced growth factors (50 μl per injection, BD Biosciences San Jose, CA, USA). Cells from a confluent 100-mm dish were injected subcutaneously into the hind leg of 7-week-old BALB/c nude mice (ORIENT Bio, Gyeonggi, South Korea) [2]. At 12 weeks after injection, teratomas were collected from the injection site, dissected, and fixed overnight in 4% formaldehyde. The teratoma tissues were then processed, embedded in paraffin wax, sectioned at 5 μm, and analyzed by hematoxylin and eosin (H&E) staining.

### Induction using conditioned medium with and without stretching

To explain the effects of equiaxial stretching on colonies, we acquired and used conditioned medium rather than fresh medium. Conditioned medium was collected after 24 h of culture, and the medium was replaced daily in all of the experiments. Three groups were set according to medium source and culture: conditioned medium from unstretched cells (Group FM) and cultured without stimulation (Group A), the same medium as used in Group A and cultured with stimulation (8% strain for 25 s/125 s/120 min per day) (Group B), and conditioned medium from stretched cells (Group FM8%4D) and cultured without stimulation (Group C). Cells in these three groups were cultured for 4 days, and medium was replaced daily (Table 2).

**Table 2:** Three groups set to investigate the effect of conditioned media

	Conditioned media from FM8%4D	Cultured with FM8%4D
Group A	X	X
Group B	X	O
Group C	O	X

### Statistical analysis

One-way analysis of variance (ANOVA) was performed using the SPSS Statics 18.0 software (SPSS Inc., Chicago, IL, USA). When ANOVA indicated a significant difference among groups the difference was then evaluated using the least-significant difference (LSD) test. All data are presented as the means ± standard deviation (SD). In all analyses, *p* < 0.05 was taken to indicate statistical significance.

## Results

**Fig. 3:**
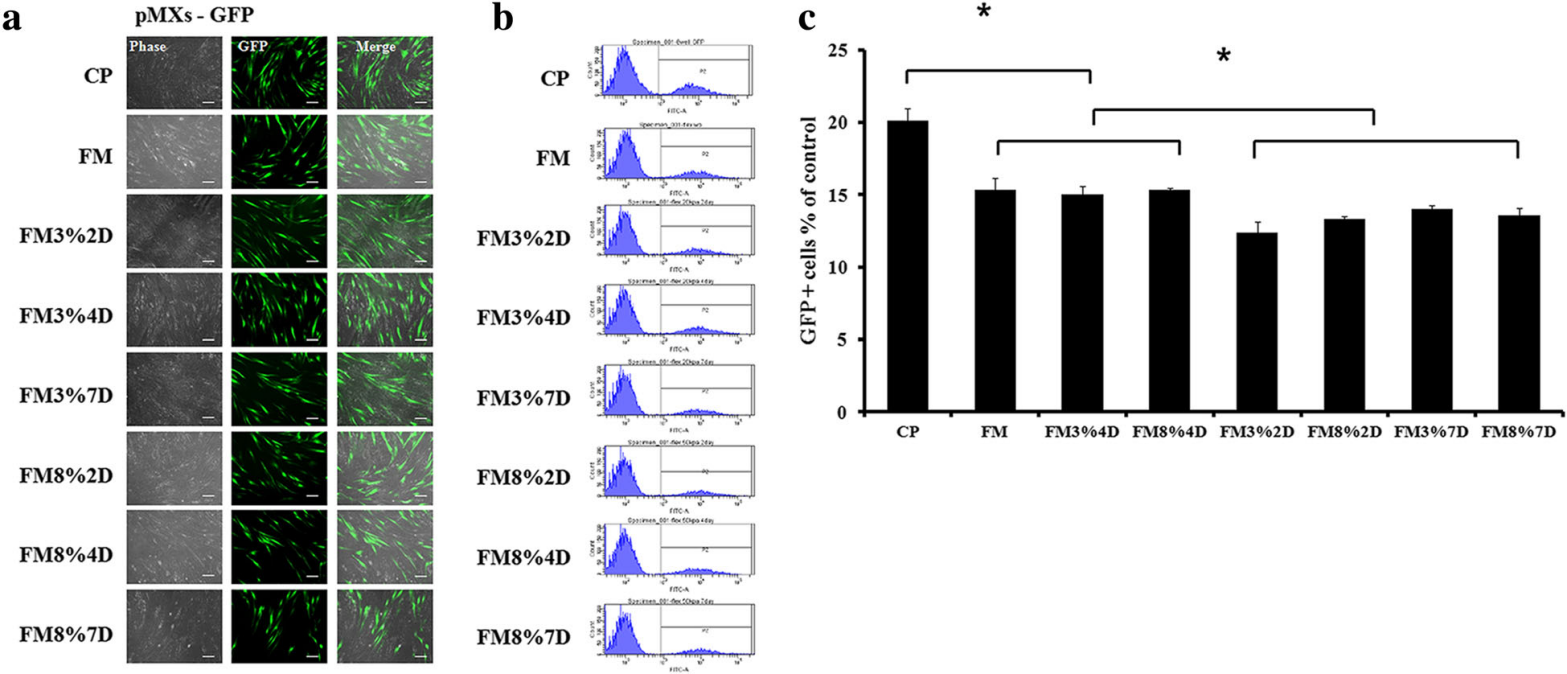
Confirmation and efficiencies of retro-GFP virus infection of dermal fibroblasts. **a** Observation of GFP-positive cells in the eight groups at day 7: phase images of virus-infected cells (*left row*), GFP-positive cells (*middle row*), and merged images (*right row*). S*cale bar* = 100 μm. **b** FACS analyses and **c** percentage of cells that were GFP-positive at day 7. *p < 0.05. *CP* commercial plates, *D* days, *FM* flexible membranes, *GFP* green fluorescent protein

Figure 3a and b show the results of retro-GFP virus infection efficiency in the eight groups at day 7, just before the infected cells were seeded on feeder cells (STO). Qualitative and quantitative analyses (Fig. 3b and c), using GFP fluorescence images and FACS, revealed lower infection efficiencies when the cells were on flexible membranes than when on commercial culture plates. However, mechanical stretching can only be provided with cells on flexible membranes. All of the flexible membrane groups had significantly lower infection efficiencies than the CP group. However, the FM, FM3%4D, and FM8%4D groups had significantly higher infection efficiencies than the other flexible membrane groups. From these results, we chose four groups (CP, FM, FM3%4D, and FM8%4D) for further investigation, and introduced four reprogramming factors (OSKM) to prepare iPSCs.

**Fig. 4:**
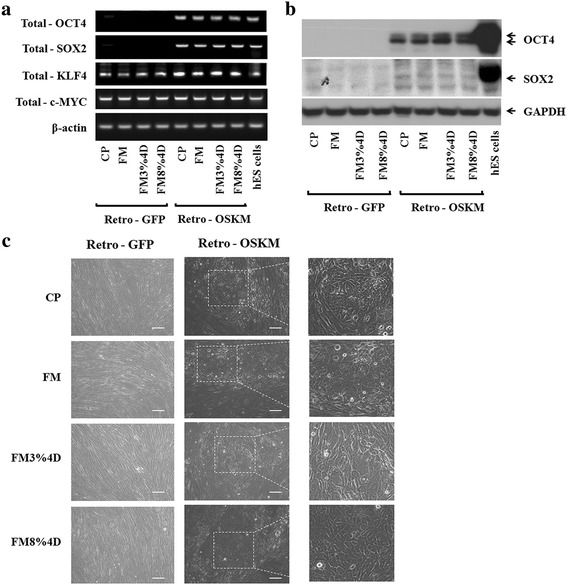
Generation of iPS cell with retroviral GFP and OSKM transduction. **a** Gene expression in the CP, FM, FM3%4D, and FM8%4D groups by RT-PCR at day 7. **b** Western blot analysis of OCT4 and SOX2 expression at day 7. **c** Retroviral OSKM transduction cell morphology change: observation of retroviral GFP infection cell morphology (*left row*), retroviral OSKM infection cell morphology (*middle row*), and magnified image (*right row*). *Scale bar* = 100 μm. *CP* commercial plates, *D* days, *FM* flexible membranes, *GFP* green fluorescent protein, *Retro-OSKM* pMXs-OCT4, pMXs-SOX2, pMXs-KLF, pMXs-c-MYC

At day 7, just before the infected cells were seeded on feeder cells, RT-PCR and Western blot analyses were performed. OCT4- and SOX2-related genes (Fig. 4a) and proteins (Fig. 4b) were expressed in all of the four groups. It is worth noting that KLF4 and the c-MYC gene are inherently present in dermal fibroblasts.

Figure 4c shows morphological observations at day 7. The cells still showed similar morphologies to those of fibroblasts infected with the GFP virus only. However, they tended to exhibit smaller, endothelial-like shapes when they were infected with the four reprogramming factors, OSKM. They also seemed to grow closer together. Overall, neither mechanical stimulation nor substrate type apparently affected the levels of gene expression and proteins or morphological changes.

**Fig. 5:**
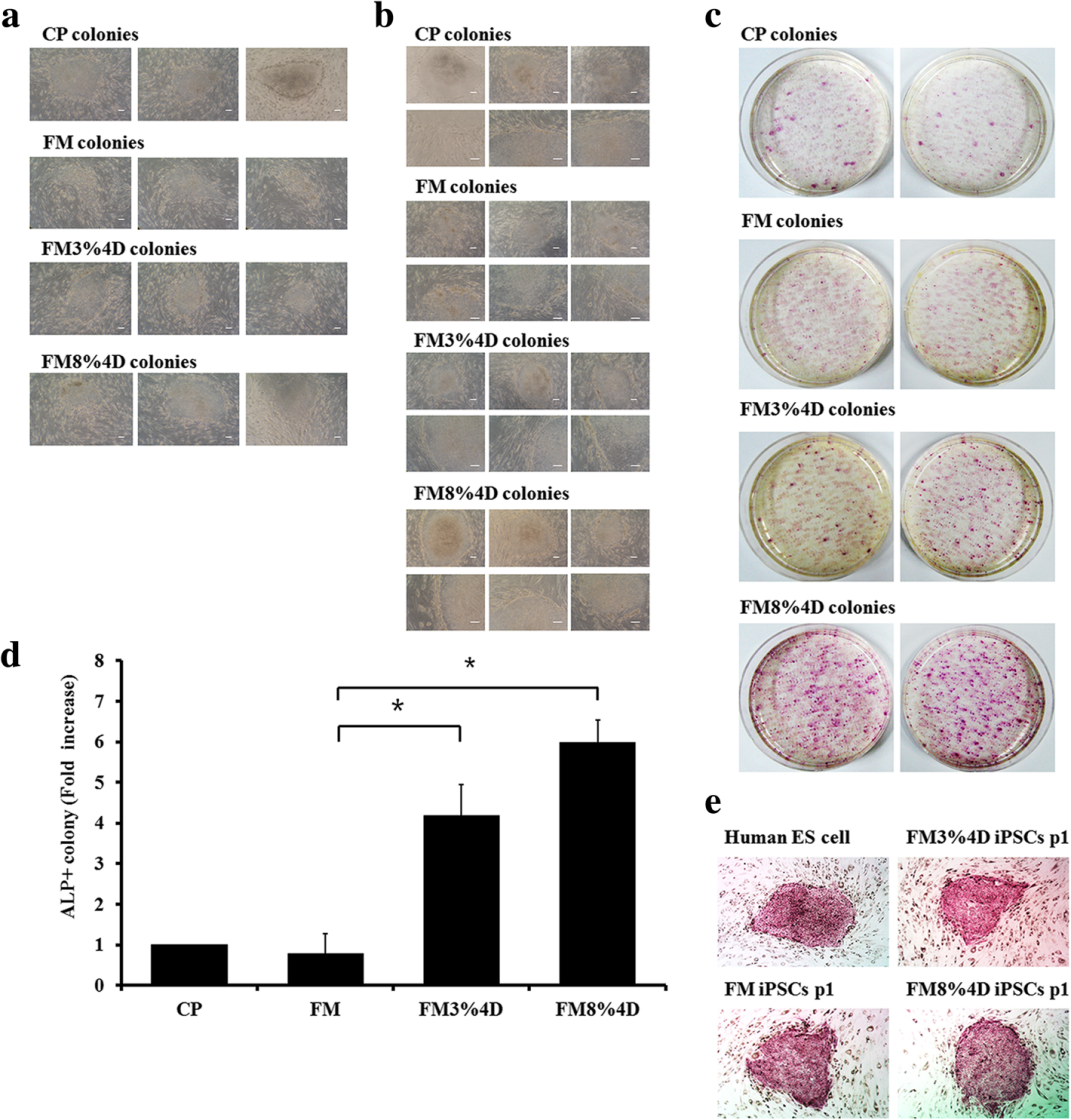
Typical morphology and ALP staining in each group **a** at day 14 (7 days after being seeded on STO) and **b** at day 21. **c** Significantly more colonies were obtained in group FM8%4D than in the other groups, especially group CP. **d** The number of ALP^+^ colonies in each group was measured using Image J and is expressed as the fold-ratio compared to group CP. **e** ALP staining showed that the morphology of iPSCs in the three groups (CP, FM3%4D, and FM8%4D) was similar to that of human ES cells. *p < 0.05. *ALP* alkaline phosphatase, *CP* commercial plates, *D* days, *ES* embryonic stem, *FM* flexible membranes, *iPSC* induced pluripotent stem cell

Typical morphologies of transduced cells in the four groups at days 7 and 14 after seeding onto STO are shown in Fig. 5a and b. At day 7, a human ES cell-like morphology was observed in all groups. This trend was also observable at day 14. A clear edge of the colony was observed in the CP group. Groups FM3%4D and FM8%4D also showed clear edges, while group FM did not.

At 14 days after being placed on STO cells, all of the transduced dermal fibroblasts on the plate (diameter = 60 mm) in human ES cell medium were fixed and stained for ALP. Fewer colonies were stained in group FM than in group CP. However, when they were stretched, many more colonies were stained: at least three- and five-times more colonies were stained in groups FM3%4D and FM8%4D than in group FM, respectively (Fig. 5c and d). Even after subculture, all were ALP-positive, similar to human ES cells (Fig. 5e). These results indicate that higher efficiency in reprogramming can be obtained with mechanical stretching even though the infection efficiency in the stretched groups was lower than that in group CP (Fig. 3d).

**Fig. 6:**
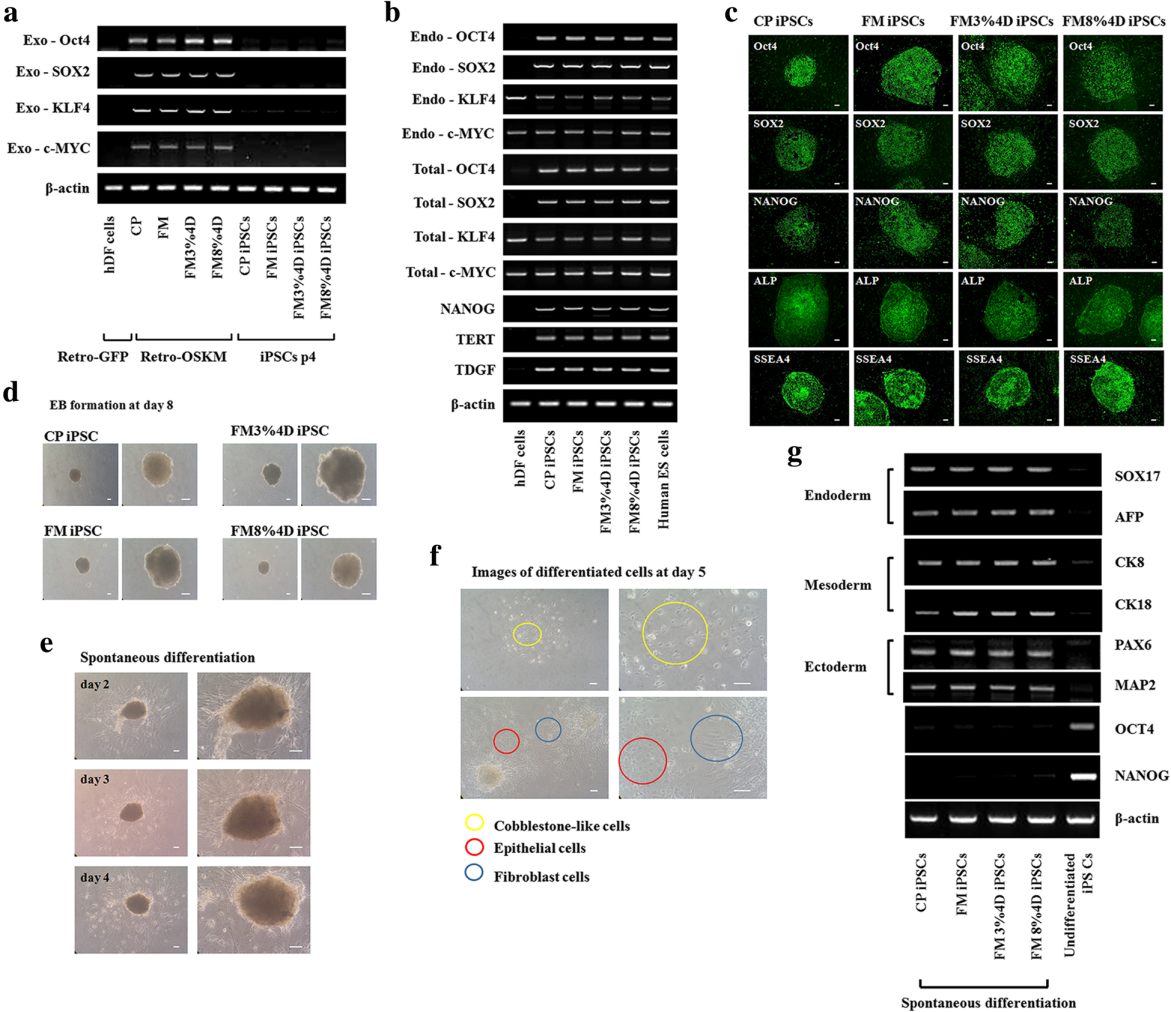
Characterization of iPSCs. **a** Confirmation of exogenous OCT4, SOX2, KLF4, and c-MYC transduction. **b** Expression of iPSC-related markers at passage 4 (OCT4, SOX2, NANOG, TERT, and TDGF). **c** Confirmation of the differentiability of iPSCs by immunostaining for OCT4, SOX2, NANOG, ALP, and SSEA. *Scale bar* = 100 μm. **d** Formation of embryonic bodies at passage 4. **e**, **f** Morphology of spontaneously differentiated cells after being cultured on dishes coated with 0.1% gelatin. *Scale bar* = 100 μm. **g** Expression of related markers to confirm three germ-layered differentiation: SOX17, AFP, CK8, CK18, PAX, and MAP2. Confirmation of loss of stem cell markers in spontaneously differentiated cells. *ALP* alkaline phosphatase, *CP* commercial plates, *D* days, *EB* embryoid body, *Exo* exogenous, *FM* flexible membranes, *GFP* green fluorescent protein, *iPSC* induced pluripotent stem cell, *Retro-OSKM* pMXs-OCT4, pMXs-SOX2, pMXs-KLF, pMXs-c-MYC

Figure 6a shows the presence of OCT4, SOX2, KLF4, and c-MYC, which were exogenously overexpressed by the retroviruses. Before being placed on STO cells, they showed the corresponding exogenous genes, while they were not seen in iPSCs at passage 4, as expected and as reported previously [1, 19].

RT-PCR was performed again on the prepared cells at passage 4. As shown in Fig. 6b, typical markers (endo-OCT4, endo-SOX2, total-OCT4, total-SOX2, NANOG, TERT, and TDGF) of iPSCs were observed. The results of immunostaining confirmed that the prepared cells were iPSCs (Fig. 6c). This was true for all four groups (CP, FM, FM3%4D, FM8%4D), regardless of stimulation and substrate type.

A primary requirement of iPSCs is that they should have the potential to differentiate, as other stem cell types do. To evaluate the differentiation potential of the cells, iPSC colonies at passage 4 were cultured on nonadherent dishes in DMEM/F12 with 20% SR for 8 days to generate EBs. EBs were confirmed in all four groups (Fig. 6d). These bodies were found to differentiate spontaneously on gelatin-coated dishes in the same medium after 4 days in culture (Fig. 6e). Figure 6f shows their morphology to be similar to that of cobblestone-like cells, epithelial cells, and fibroblast cells. Additionally, the RT-PCR results (Fig. 6g) showed endoderm-, mesoderm-, and ectoderm-related markers, confirming that the iPSCs prepared showed pluripotent characteristics. In addition, the loss of stem cell markers was confirmed in the spontaneously differentiated cells.

**Fig. 7:**
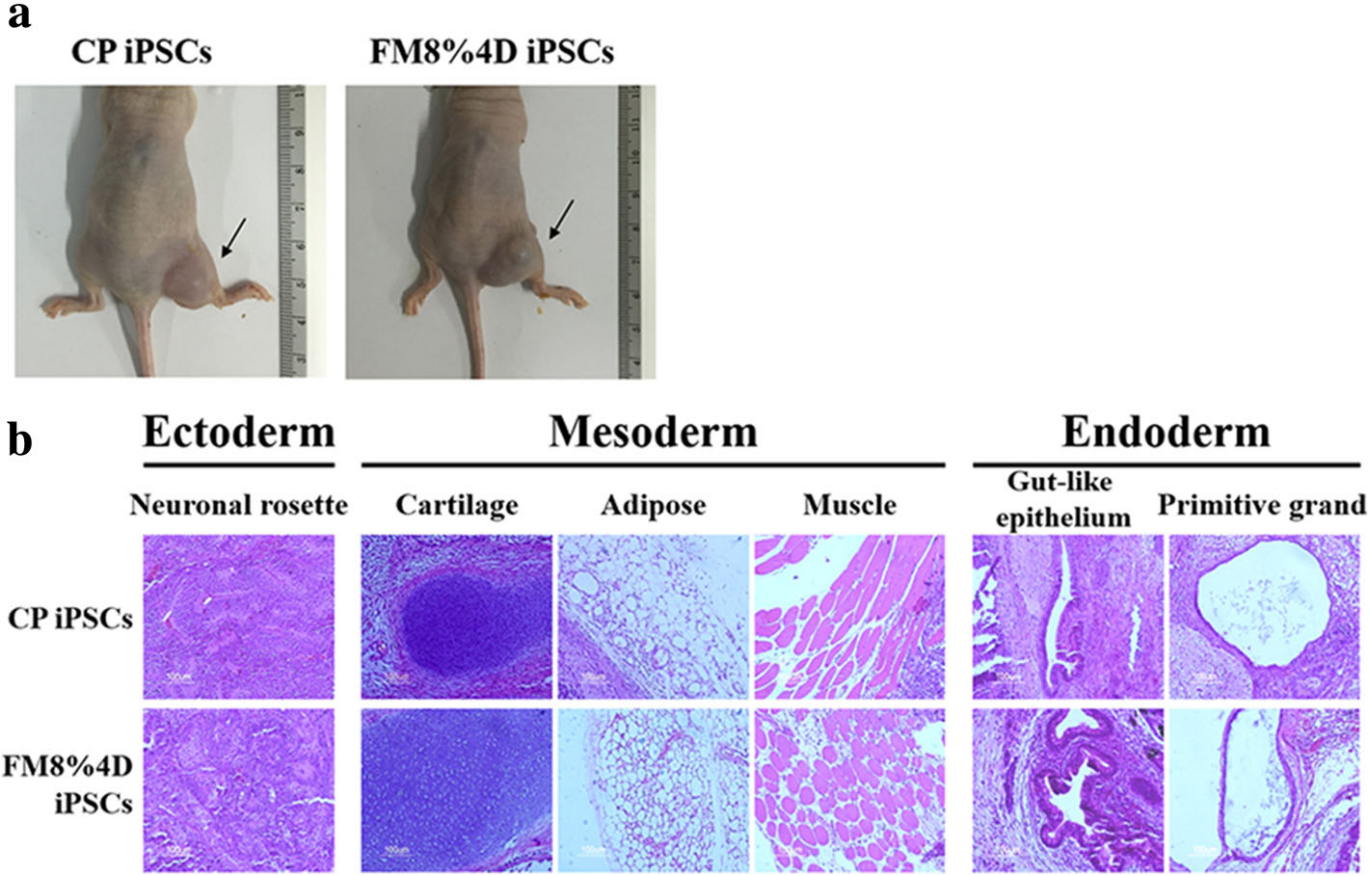
Confirmation of teratoma formation and H&E staining. **a** Teratoma formation and **b** H&E staining show distinct differentiation into an ectoderm, mesoderm, and endoderm. *CP* commercial plates, *D* days, *FM* flexible membranes, *iPSC* induced pluripotent stem cell

We also confirmed another essential characteristic, the formation of teratomas, through animal tests. For this, the iPSCs prepared (passage 8), combined with Matrigel, were injected into BALB/c nude mice (CAnN, Cg-Foxn1nu/CrljOri, 7 weeks old). At 12 weeks after injection, teratomas were confirmed (Fig. 7a). H&E staining was performed on the tissue. In the teratoma, we could see distinct differentiation into neural tissue (ectoderm), adipose, muscle cartilage (mesoderm), and primitive grand and gut-like epithelium (endoderm) (Fig. 7b). These results indicate that our hiPSCs had been reprogrammed into a pluripotent state.

**Fig. 8:**
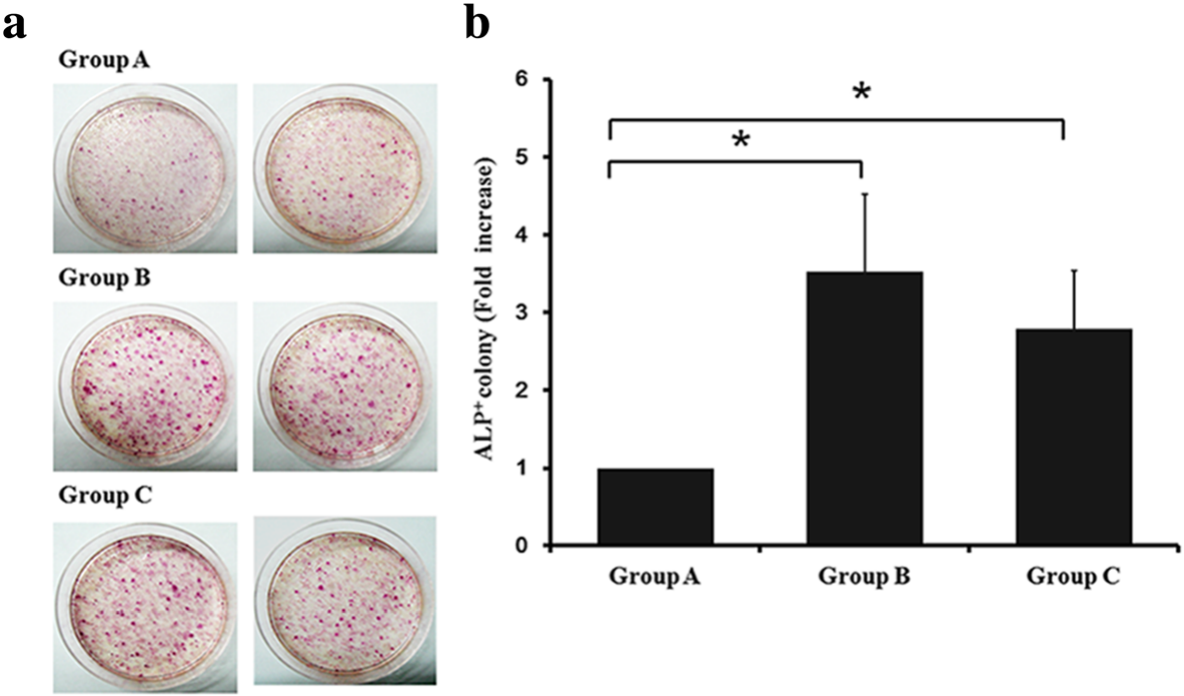
Results of experiments with conditioned medium. **a** ALP staining of colonies in each group. **b** Number of ALP^+^ colonies is presented as fold-ratios compared to group A. *p < 0.05. *ALP* alkaline phosphatase

Finally, we tried to assess clues underlying the enhanced colony formation upon application of mechanical stimulation. The entire experimental group is shown in Table 2. When we used conditioned medium from the cells cultured without stimulation and simply cultured them, we could acquire the least number of iPSC colonies among the three groups set in this test (Group A). When we used the same conditioned medium and cultured the cells with stretching (8% strain for 25 s/125 s/120 min per day) a greater number of colonies was observed (Group B). However, fewer colonies than in Group B were obtained when the cells were cultured without stimulation in conditioned medium of stretched cells (FM8%4D) (Group C), although the difference between Groups B and C was not significant (Fig. 8a and b). From these results, it seems that some cytokines, as yet unknown, may be generated or secreted by the cells during stretching. These unknown cytokines may play roles in colony generation.

## Discussion

Since the first description of the preparation of iPSCs, many investigators have suggested various techniques or methods to improve its efficiency. Chemical compounds other than oncogenic transcription factors have been found to be effective in improving efficiency [20–24]. Moreover, biophysical factors, such as the mechanical properties and topography of substrates, have also been found to affect the preparation of iPSCs [25]. However, little has been reported regarding the preparation of iPSCs using mechanical stimuli.

To our knowledge, this is the first reported study using mechanical stimuli in the preparation of iPSCs and, in particular, in the preparation of human iPSCs. Our obtained results show the promise of this approach. Specifically, the reprogramming efficiency was increased, with more colonies formed, when mechanical stimulation was provided by, in this case, equiaxial stretching.

The efficiency of retrovirus infection (pMXs-GFP) was found to be affected by substrate stiffness, and the efficiency of group CP was higher than that of the other groups using a flexible membrane. This may be explained by the fact that most cells are more easily infected when they are dividing/proliferating. Other studies have shown that cells proliferate more when they are on stiffer rather than softer substrates [26–30]. The commercial plate used was much stiffer than the flexible membrane used in group FM. Thus, a higher infection ratio in group CP was expected than in group FM, which was confirmed.

iPSC colonies were found in every group, regardless of substrate type and stretching. However, the colonies prepared on flexible membranes without stretching were found to be unstable, with unclear edges. Moreover, their average size was smaller than in the stretched groups. While the infection efficiency was comparable to that of the groups undergoing stretching, it was much lower than that in group CP. Given that many more colonies were obtained from the stretched groups, especially group FM8%4D, the infection efficiency measured by FACS is not a useful indicator of successful preparation of iPSCs. Another important issue is the reprogramming environment after infection, including mechanical stimuli, as suggested by this study. Additionally, the colonies in group FM8%4D showed clear edges with comparable sizes and were found to be reprogrammed successfully into iPSCs through various analyses and animal tests. Thus, a role for mechanical stimulation (equiaxial strain) was confirmed in that it contributed to reprogramming efficiency rather than to infection efficiency.

Many investigations of the effects and corresponding mechanisms of mechanical stimuli on stem cells have been reported. However, most showed the resulting effects rather than clarifying the underlying mechanisms, stating simply that mechanical stimuli can affect gene expression, protein synthesis, cell proliferation, and other cellular activities [31–33]. The experiments performed in this study provide information to explain the effect of mechanical stretching on the formation of more colonies. When we attempted to separate the effect of conditioned medium and mechanical stimulation, mechanical stimulation apparently played the dominant role in producing a greater number of colonies: more colonies were formed in Groups B and C than in Group A. However, we cannot rule out auto- or paracrine effects on reprogramming when cells were stimulated.

In investigating the detailed mechanisms based on signaling pathways, some previous studies [34–38] reported only on mesenchymal stem cells or various somatic cells, investigating MAPK/ERK and Rho signaling and how they affected differentiation and cell phenotypes. Regarding reprogramming in iPSCs, there are no reports on the basic mechanism(s) and signaling pathway(s) affected by mechanical stimuli. Indeed, even in this study, we were unfortunately not able to clarify the basic mechanisms underlying the role of mechanical stimuli in reprogramming processes.

## Conclusions

This is the first reported study to investigate the effects of mechanical (equiaxial) stretching on the preparation of human iPSCs. While there was no positive effect on virus infection, the stretched groups formed a greater number of iPSC colonies than the unstretched groups. This indicates that mechanical stretching has a positive effect in reprogramming. Additionally, experiments using conditioned media from the stretched and unstretched groups indicated that mechanical stimulation plays an important role in preparing human iPSCs, although the mechanical stretching conditions in this study were limited. This study suggests a new paradigm in human iPSC studies. Further studies to examine the effects of various mechanical conditions on long-term stability and animal experiments should be performed.

## Abbreviations

ALP: Alkaline phosphatase
ANOVA: Analysis of variance
bFGF: Basic fibroblast growth factor
BSA: Bovine serum albumin
CP: Commercial plates
D: Days
DMEM/F12: Dulbecco’s modified Eagle’s medium/Ham’s F-12
DMEM: Dulbecco’s modified Eagle’s medium
EB: Embryoid body
EDTA: Ethylenediaminetetraacetic acid
ES: Embryonic stem
EtBr: Ethidium bromide
FACS: Fluorescence-activated cell sorting
FBS: Fetal bovine serum
FGM-2: Fibroblast growth medium-2
FM: Flexible membranes
GFP: Green fluorescent protein
H&E: Hematoxylin and eosin
iPSC: Induced pluripotent stem cell
KLF4: Kruppel-like factor 4
NEAA: Non-essential amino acids
OCT4: Octamer-binding transcription factor 4
ONSL: OCT4, NANOG, SOX2, and Lin28
OSKM: OCT4, SOX2, KLF4, and c-MYC
PBS: Phosphate-buffered saline
PVDF: Polyvinylidene fluoride
Retro-OSKM: pMXs-OCT4, pMXs-SOX2, pMXs-KLF, pMXs-c-MYC
RT-PCR: Reverse-transcription polymerase chain reaction
SOX2: Sex determining region Y-box 2
SR: Knockout serum replacement
VPA: Valproic acid
